# Systematic Approach to Deep Plane Facelift

**DOI:** 10.1007/s00266-025-04793-0

**Published:** 2025-03-25

**Authors:** Boris M. Ackerman, Nirav B. Savalia

**Affiliations:** 1 Newport Beach, CA USA; 2 Savalia Plastic Surgery, 361 Hospital Road, Suite 530, Newport Beach, CA 92663 USA

**Keywords:** Deep plane facelift, Composite cheek flap, Facial nerve, Monobloc

## Abstract

**Abstract:**

The senior author has developed a systematic approach to performing Deep Plane Facelift, which allows the procedure to be performed in a safe, efficient and reproducible fashion. This approach evolved over many years of facial rejuvenation surgery practice. The senior author was introduced to Deep Plane Facelift in Dr. Sam Hamra’s operating room over 20 years ago. The technique has three distinct dissection steps, and a unique suture fixation method. This method allows for performing Deep Plane Facelift in an efficient and safe fashion. This technique has some novel elements: strict compartmentalized dissection sequence; lateral orbicularis muscle is incorporated into the composite cheek flap; treat the mobilized composite cheek and neck flap as a “monobloc”; the cheek suspension starts at the superior most aspect of the composite cheek flap; the cheek flap is advanced in a more superior vector than traditionally described; the cheek flap is held in pre-determined position with a hook, under moderate tension during suture fixation. The manipulation of the neck is beyond the scope of this article; it can be done by any of the described methods, as indicated by the patient’s individual anatomy. Having received positive reports from visiting surgeons that have learned this technique from the senior author, we hope that others will find this technique beneficial. We will present a few representative long-term results.

**Level of Evidence V:**

This journal requires that authors assign a level of evidence to each article. For a full description of these Evidence-Based Medicine Ratings, please refer to Table of Contents or online Instructions to Authors www.springer.com/00266.

**Supplementary Information:**

The online version contains supplementary material available at 10.1007/s00266-025-04793-0.

## Introduction

Facelift techniques have evolved significantly, thanks to the contributions of pioneering surgeons aiming for natural and lasting results. In the 1970s, Dr. Tord Skoog introduced the concept of a composite flap, addressing facial sagging and excess skin [1]. Around the same time, Drs. Vladimir Mitz and Martine Peyronie advanced anatomical research on facial structures, particularly the Superficial Musculo-Aponeurotic System (SMAS) [2]. The 1990s saw Dr. John Owsley focus on malar fat pad repositioning for correcting nasolabial folds, while Dr. Sam Hamra described a composite facelift [3, 4]. Dr. Bryan Mendelson has published extensive anatomic studies and descriptions of the facial compartments and relevant neurovascular anatomy, advancing our understanding of facial anatomy [5–9]. Recently, Dr. Andrew Jacono has incorporated innovative techniques and helped popularized the deep plane facelift by demonstrating excellent results with an excellent description of his methods [10]. This progression highlights a dedication to refining surgical methods and improving patient outcomes.

The authors believe the Deep Plane Facelift represents the pinnacle of facial rejuvenation surgery, involving the complete release of the cheek retaining ligaments. This allows for correction of the mid-cheek, resulting in a more meaningful rejuvenation compared to earlier techniques. Despite its benefits, many contemporary plastic surgeons are hesitant to perform this technique due to concerns about potential injury to the Zygomatic and Buccal branches of the Facial Nerve. The authors present a unique systematic, step-by-step approach that distinguishes itself from other modern methods, enabling the Deep Plane Facelift to be performed efficiently, reproducibly, and safely; additionally, the authors present a few novel technical elements that have not been previously described in the literature

## Surgical Technique

In the preoperative phase, the patient is marked for a standard rhytidectomy incision, which preserves the sideburns/temporal hair tuft (Video 1). The incision follows the tragal edge, moves along the postauricular crease, extends to the occipital hairline, and continues inferiorly along the hairline. A line is also drawn from the lateral orbital rim to the gonial angle to indicate the deep plane dissection entry point.

After general anesthesia induction, typically with an LMA, the face is tumesced using a roller pump and a diluted solution of 500 cc of normal saline, 100 cc of 1% lidocaine with epinephrine 1:100,000, and 1 gram of tranexamic acid. The subcutaneous cheek skin flap is elevated to one cm medial to the deep plane entry point, as indicated by the markings. The subcutaneous dissection is meticulous, maintaining a uniform skin flap. The neck skin flap is elevated a few centimeters medial to the anterior border of the sternocleidomastoid muscle.

**Fig. 1:**
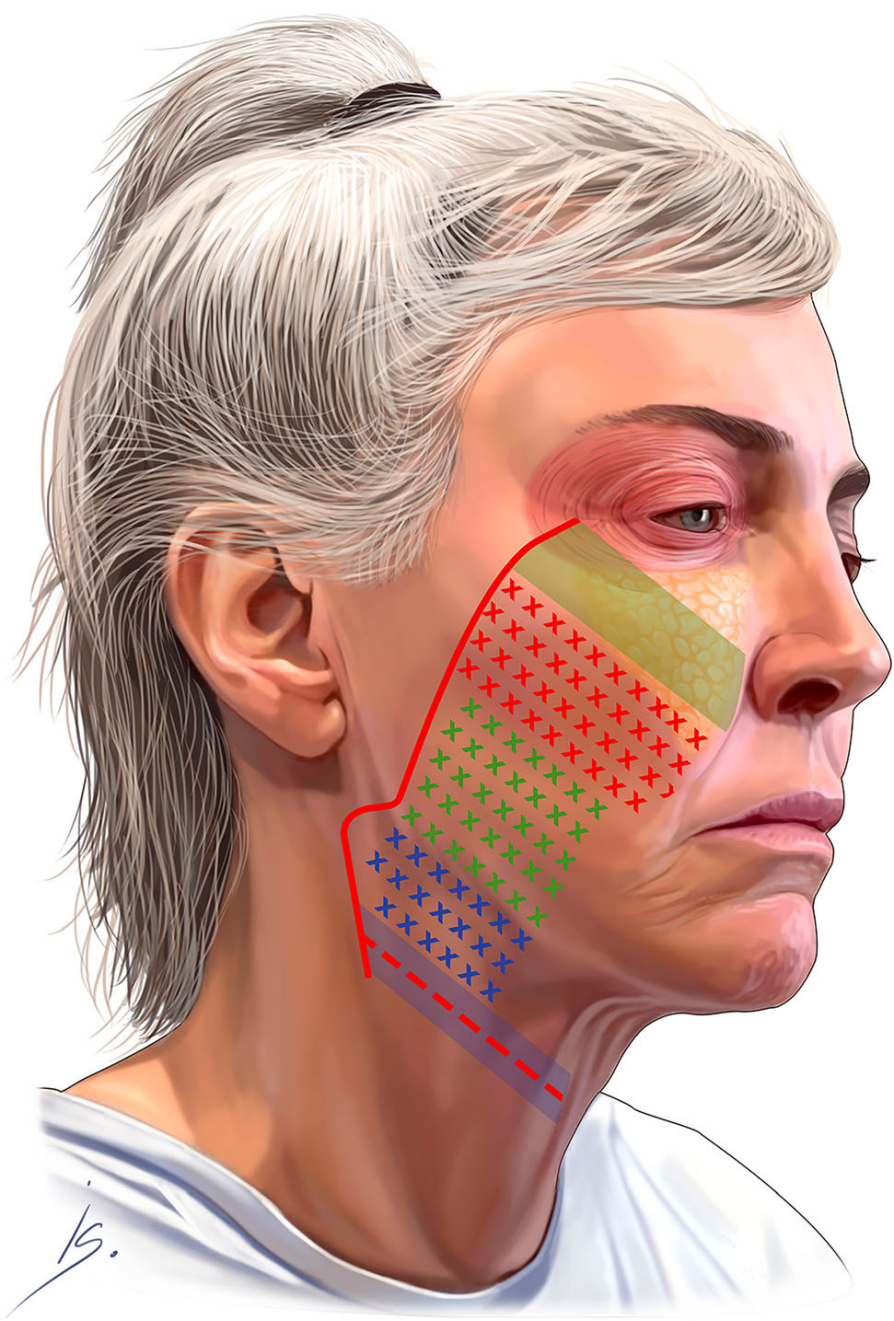
Dissection sequence. Green solid stripe: prezygomatic dissection. Green cross hatches: premasseter dissection. Red cross hatches: zygomatic ligament complex dissection. Blue cross hatches: sub-platysma dissection. Solid blue line: sub-platysma tunnel. Dashed red line: transverse transection of platysma

Knowledge of the anatomy of facial structures such as muscles, nerves, salivary glands, ducts, and fat pads, along with the branches and course of the facial nerve, is crucial [11]. The facial nerve branches are protected within the parotid gland and, after exiting, course below the deep investing fascia to innervate the mimetic muscles. The skin marking from the lateral orbit to the gonial angle is transposed onto the deep tissues, and the deep plane is sharply entered with a scalpel. The dissection proceeds in three parts (Fig. 1 and Video 2)

### Step 1: Prezygomatic and Premaxillary Space Dissection (Video 3)

The prezygomatic space is dissected first [9]. The entry into the space begins by sharply elevating the lateral orbicularis oculi and dissecting just superficial to the pre-periosteal fat overlying the zygoma, using Metzenbaum scissors. The frontal branch is well deep and lateral to this dissection plane but should be considered and protected if cranially or laterally extending the dissection. The dissection proceeds medially over the malar eminence on top of the pre-periosteal fat, spreading with progressively larger Mayo scissors to the nasolabial fold, thus opening the premaxillary space. This avascular plane allows a rapid, nearly bloodless dissection, and is similar to what has been initially described by Owsley [3] and coined the F.A.M.E. procedure by Aston [12]. An important and key difference is that this avascular plane is deep to the orbicularis oculi rather than superficial to it, and allows for unimpeded entry into the prezygomatic space [9]. The surgeon will feel resistance at the superior boundary of the space defined by the retaining orbital ligament, and inferiorly, once the zygomatic ligaments are reached. At this point, proceed to the next step, leaving the zygomatic ligaments intact.

### Step 2: Premasseter Space Dissection (Video 4)

Next, the premasseter space is elevated using small Metzenbaum scissors in a spreading motion. The dissection starts at the gonial angle, where the platysma muscle transitions into SMAS layer. This maneuver ensures that the dissection is proceeding in the proper plane. The dissection proceeds over the masseter muscle and extends medially, taking care to avoid inadvertent injury to the marginal mandibular branch by hugging the undersurface of the platysma and limited medial extent of dissection to the anterior border of the masseter muscle.

### Step 3: Zygomatic Ligament Complex Dissection (Video 5)

**Fig. 2:**
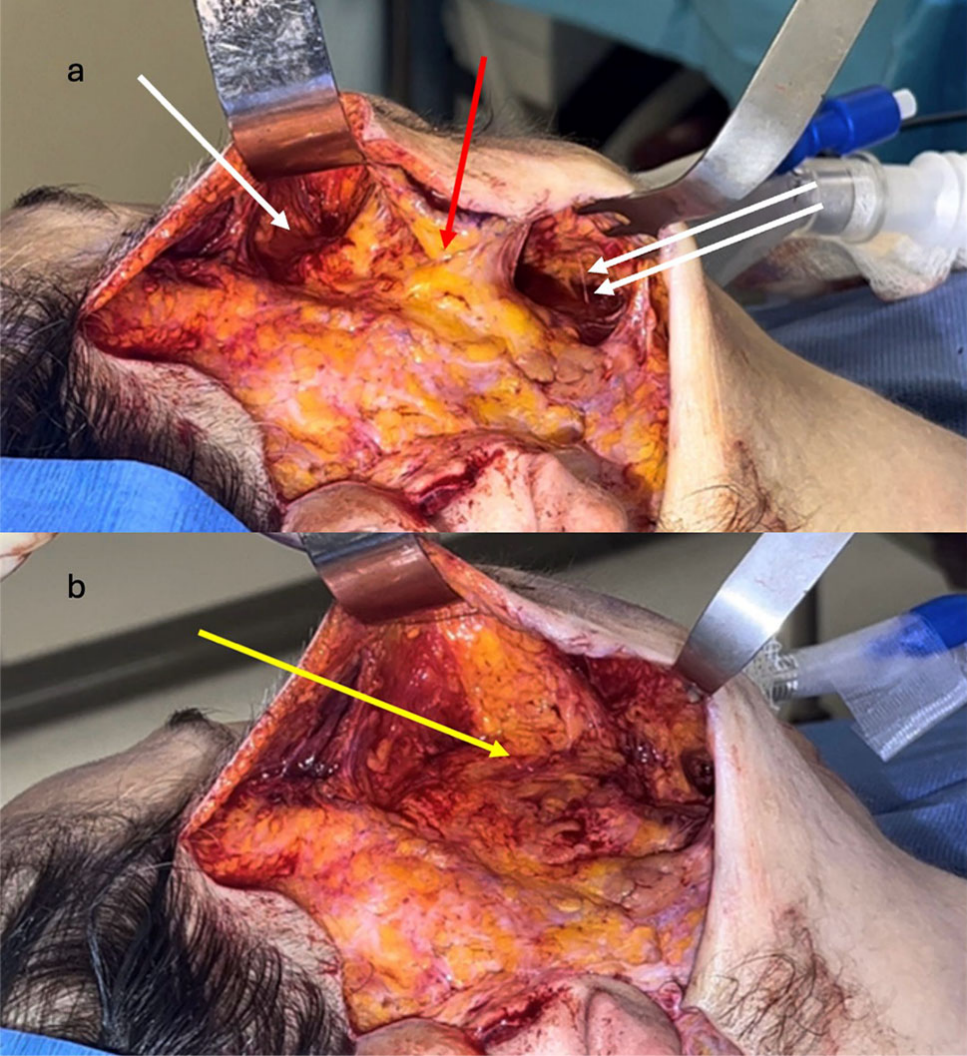
The top image (**a**) shows the prezygomatic/premaxillary space (single white arrow) and the premasseter space (double white arrow) opened with retractors in place. Traction on the retractors allows easy identification and dissection of the zygomatic ligaments (red arrow). The bottom image (**b**) show the fully released zygomatic ligaments with the plane opened medially to the nasolabial fold (yellow arrow)

Dissecting the zygomatic ligament complex (McGregor’s Patch) is the most challenging step [13, 14]. The zygomatic ligaments are isolated, tented, and held under moderate tension with a malleable retractor in the premaxillary space and an Ackerman Facelift Retractor in the premasseter space (Fig. 2). The dissection, guided by visual and tactile clues, differentiates ligamentous bands from nerve branches. The ligamentous bands are incrementally divided medially, from superior to inferior, with visualization aided by the previously completed dissection of the prezygomatic space. Complete release of the zygomatic ligaments is followed by further medial dissection towards the nasolabial fold, staying just superficial to the zygomaticus major and minor muscles (Fig. 2). This maneuver serves to fully connect the previously dissected prezygomatic space, premaxillary space and premasseter space, and fully mobilize the malar fat pad.

**Fig. 3:**
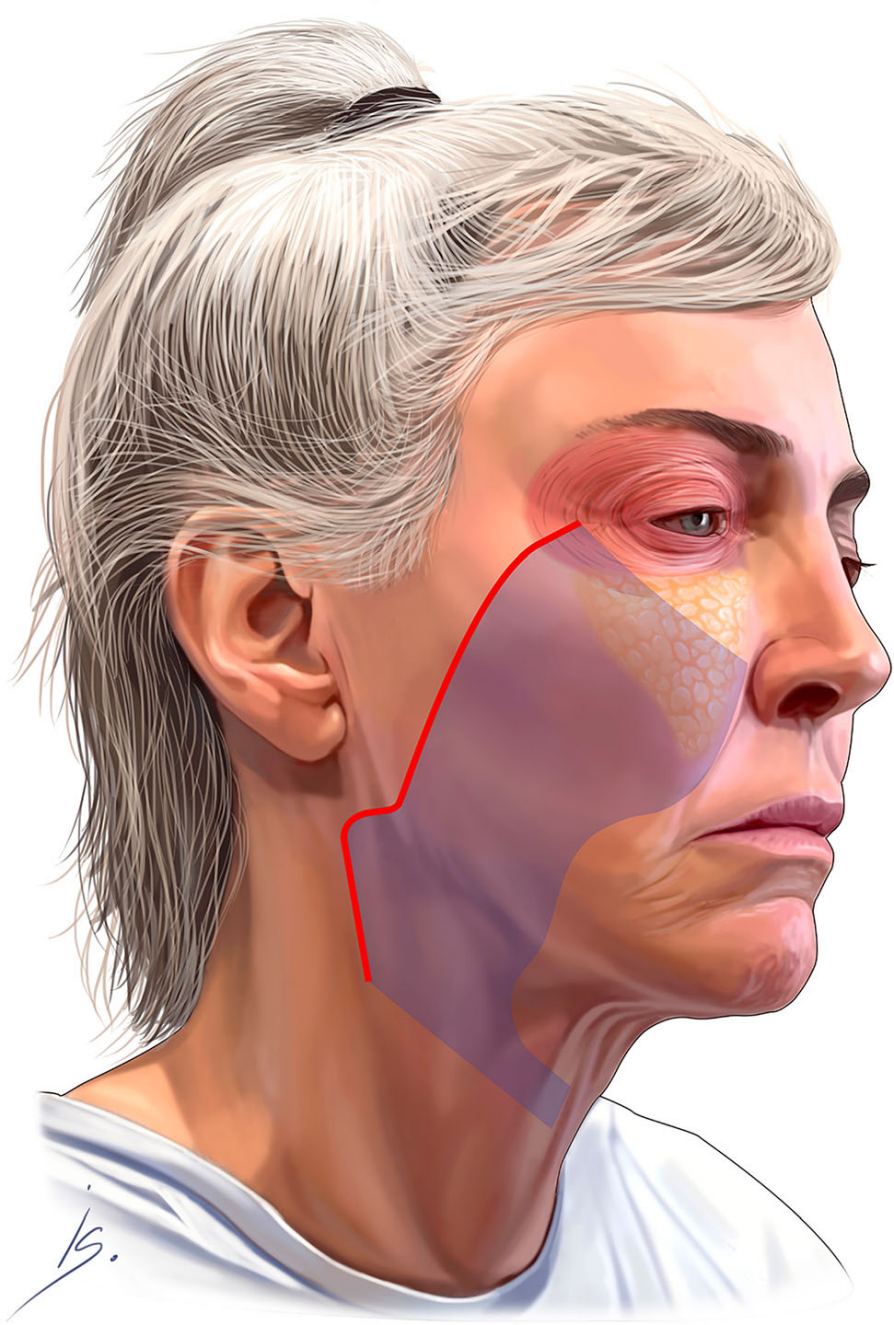
Depicts the cheek and neck tissue complex—the “monobloc”

**Fig. 4:**
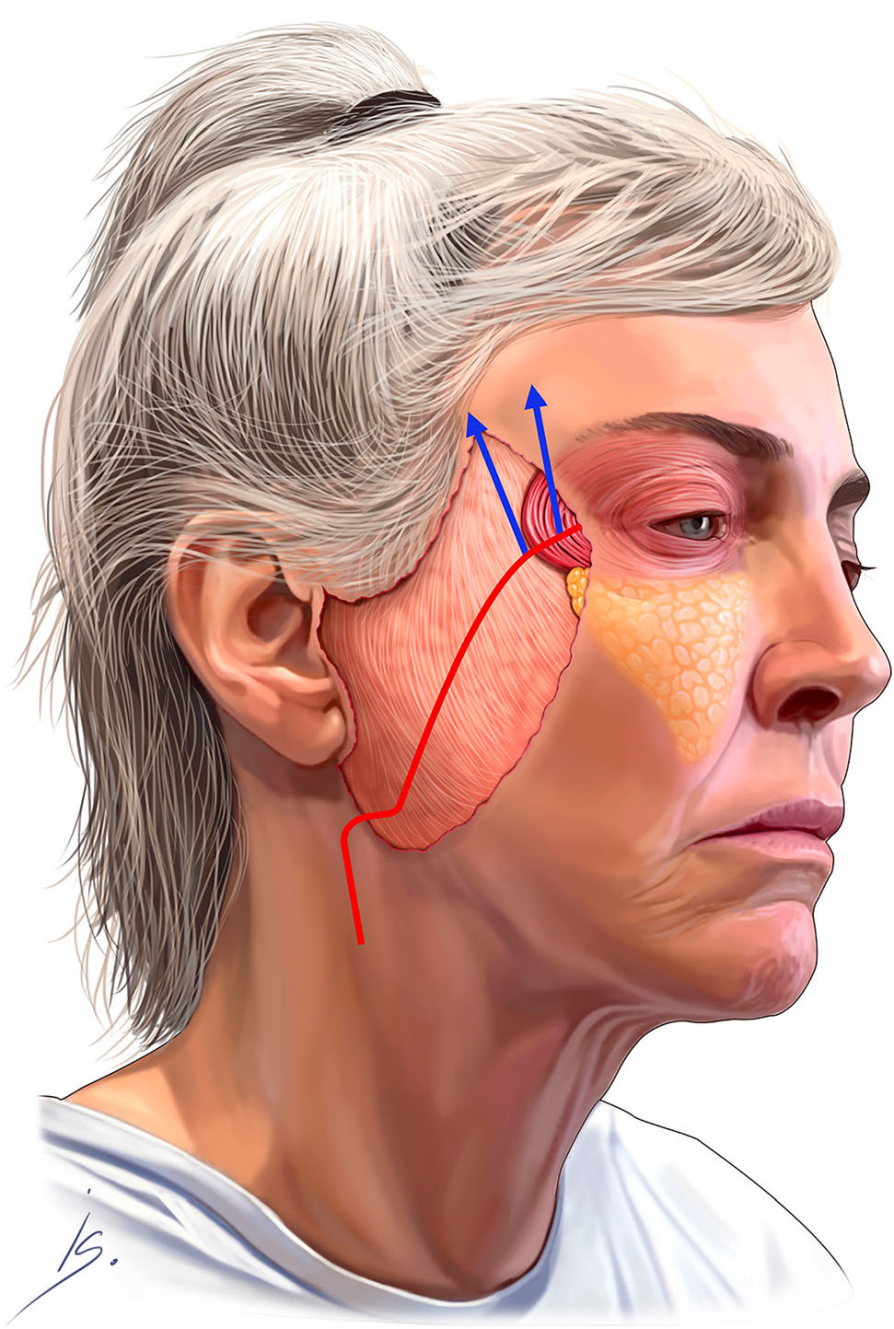
This image depicts the orbicularis muscle incorporated into the composite cheek flap; the blue arrows show the vector of “monobloc” elevation

The premasseter space dissection continues inferiorly in the sub-platysma tissue plane. The platysma is transected transversely at the level of the thyroid cartilage. After all the retaining ligaments in the cheek and the neck are released, the cheek and neck composite tissue complex (the “Monobloc”) is fully mobile and ready for fixation (Fig. 3). The fixation must start superiorly, and the desired vector is typically 10–15 degrees more vertical than the vector paralleling the zygomaticus major muscle. The first key suture (2-0 Vicryl mattress suture) is pre-placed in the superior most part of the cheek flap; it is a spanning suture over the Pitanguy’s line, to protect the frontal branch. The cheek flap is positioned vertically, and held in position under moderate tension, while the pre-placed suture is secured to the deep temporal fascia (Fig. 4, Video 6). Next, 3-4 additional sutures are placed, in identical fashion, along the length of the cheek flap. The entire cheek fixation is reinforced with a running 3-0 Vicryl suture (Video 7). None of the tissue in the cheek is trimmed, the SMAS layers are stacked on top of each other, adding bulk to the lateral cheek.

**Fig. 5:**
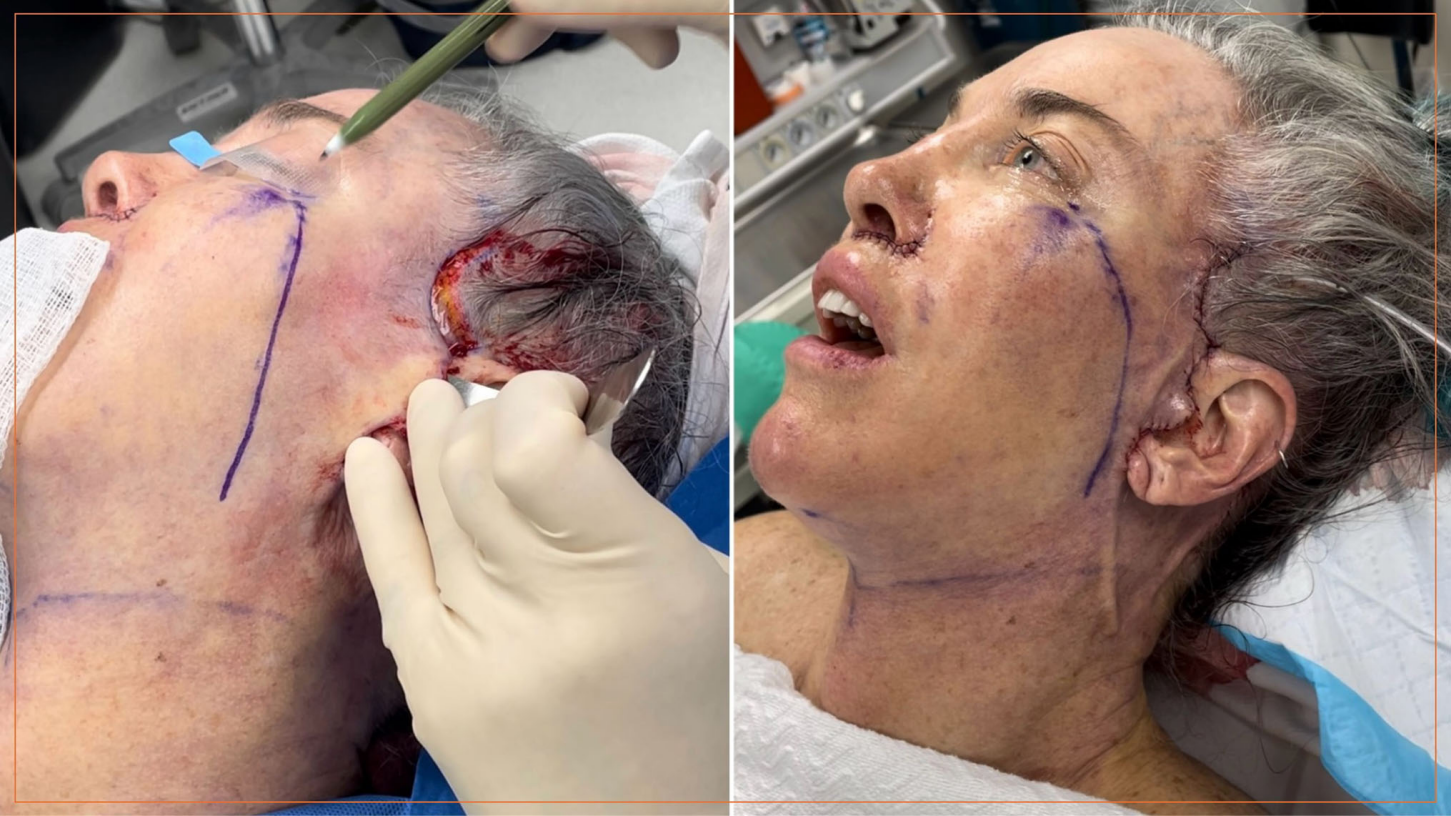
The image on the left shows the line of entry for the deep plane as marked on the skin, and the image on the right shows the same marked line after deep plane flap fixation. The marked line is now markedly elevated, demonstrating the dramatic lift achieved after deep plane mobilization and fixation

This approach produces dramatic rejuvenation of the entire cheek, creating a youthful cheek ogee. Figure 5 illustrates this transformation; the diagonal line on the cheek in the pre-fixation image is dramatically transformed in the post-fixation image; the line moves superiorly and changes from straight to a curved configuration. This illustrates the change of the cheek curves, the cheek ogee.

## Discussion

The modern deep plane facelift technique has its origin with the pioneering work of Dr. Sam Hamra. Dr. Hamra’s method uses vertical vector to reposition the mid-cheek via the orbicularis oculi muscle, accessed through the lower blepharoplasty incision. Our technique to lift the mid-cheek also relies on orbicularis oculi muscle, but without the need to open the lower eyelid. Most other techniques for deep place facelifts described in the literature typically omit incorporation and elevation of the lateral orbicularis oculi due to the concerns of motor nerve injury. Recently, Cakmak et al published their cadaveric study demonstrating the safety of incorporating elevation of the lateral orbicular oculi muscle using FAME (Finger-Assisted Malar Elevation) [15, 16]. Based on this and other anatomic studies, we believe that incorporating the lateral orbicularis oculi muscle into the composite cheek flap is a safe and meaningful step [15, 17]. Furthermore, incorporating the lateral orbicularis muscle into the composite cheek flap allows for the powerful and harmonious rejuvenation of the cheek, with sustained elevation of the malar fat pad. Once the cheek and neck composite tissue complex, the “monobloc”, is fully dissected medially to the nasolabial fold, it is positioned and held in place with a skin hook in the desired position. The vector of pull of the “monobloc” is mostly superior. This maneuver re-creates the youthful cheek ogee and repositions the facial volume, often obviating the need for structural fat grafting; in addition, this maneuver also dramatically improves the jaw line by eliminating the jowls. This vertical vector is counter to current techniques that favor a vector parallel to the zygomaticus major muscle [10]. A vector paralleling the muscle is advocated to avoid disturbing the vector of pull and distorting the smile. This assumes that the muscle has remaining attachments to the overlying tissue, however, our technique completely frees the overlying tissue with a dissection that ends medially at the nasolabial fold, thus obviating any concern about distortion. This surgical approach is universally amenable to any patient that is presenting for facial rejuvenation surgery regardless of sex or age. This method is especially powerful in patients that had prior SMAS type of a facelift. The senior author has performed revision facelifts on patients that had prior Deep Plane Facelift, as well as in patients with prior bio-stimulating filler treatments (Sculptra, Radiesse); with some caution, and slow and meticulous dissection, this method can be applied safely.

**Fig. 6:**
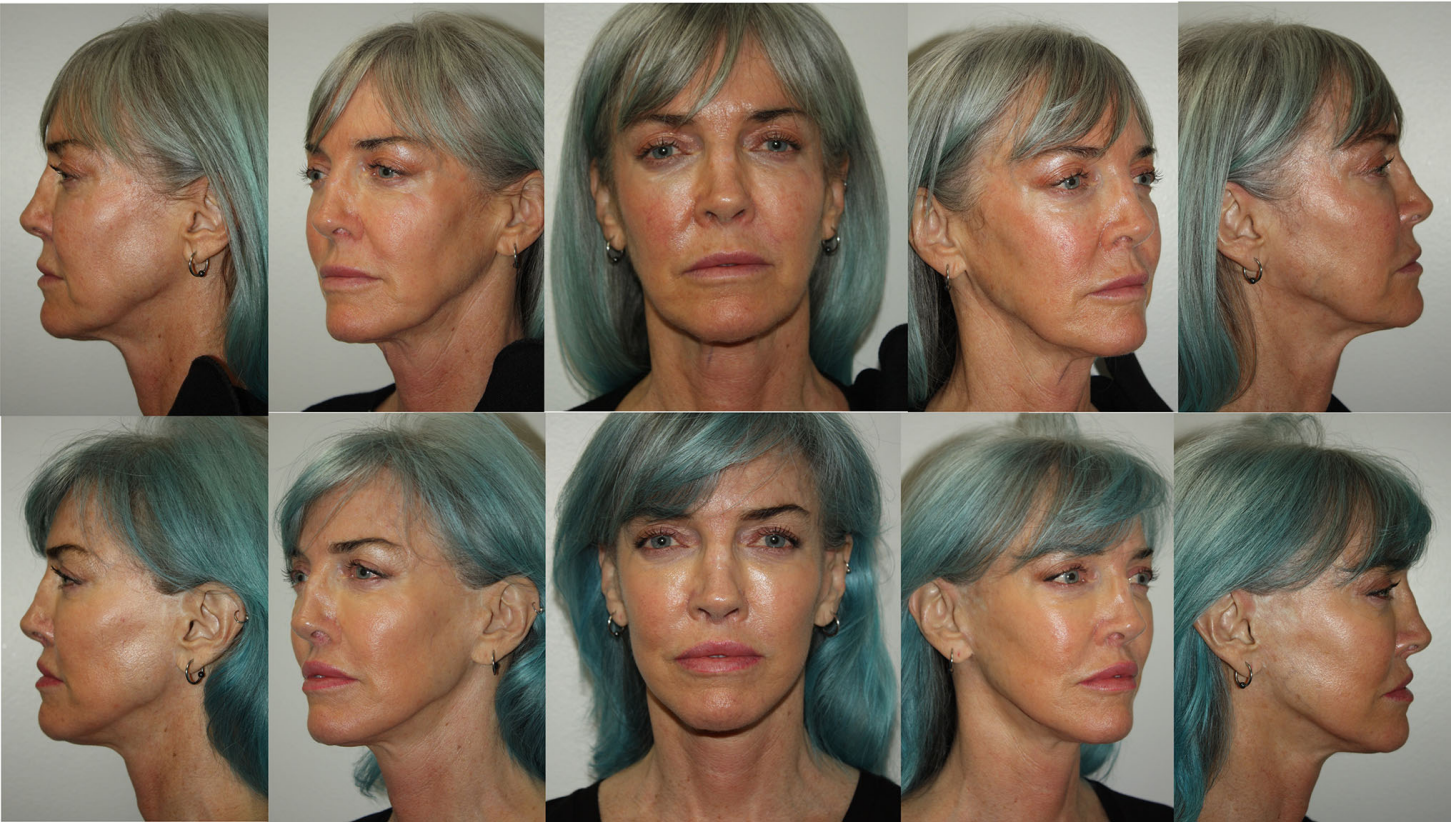
63 year old woman who is 2 years post op from deep plane facelift and subnasal lip lift only. No other procedures have been performed nor additional volume added. She is the same patient shown in Fig. 1

**Fig. 7:**
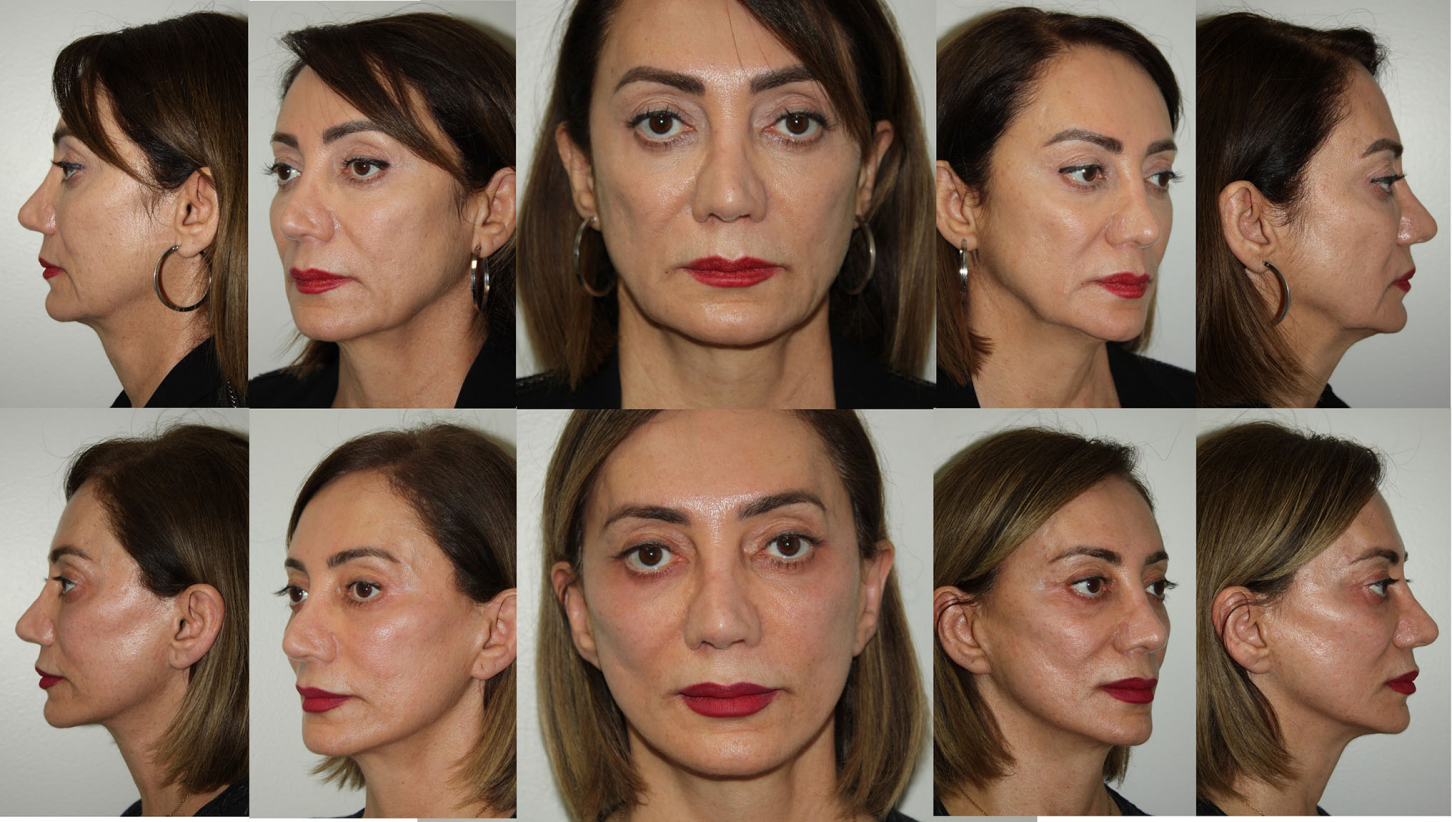
59 year old woman who is 3 years post op from deep plane facelift and upper blepharoplasty only. No other procedures have been performed nor additional volume added

**Fig. 8:**
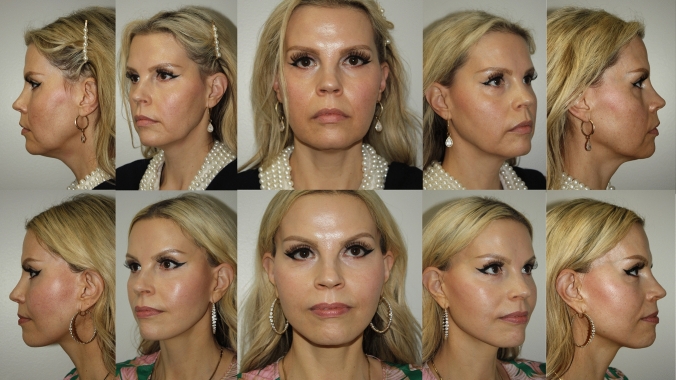
50 year old woman who is 2 years post op from deep place facelift and lower blepharoplasty only. No other procedures have been performed nor additional volume added

**Fig. 9:**
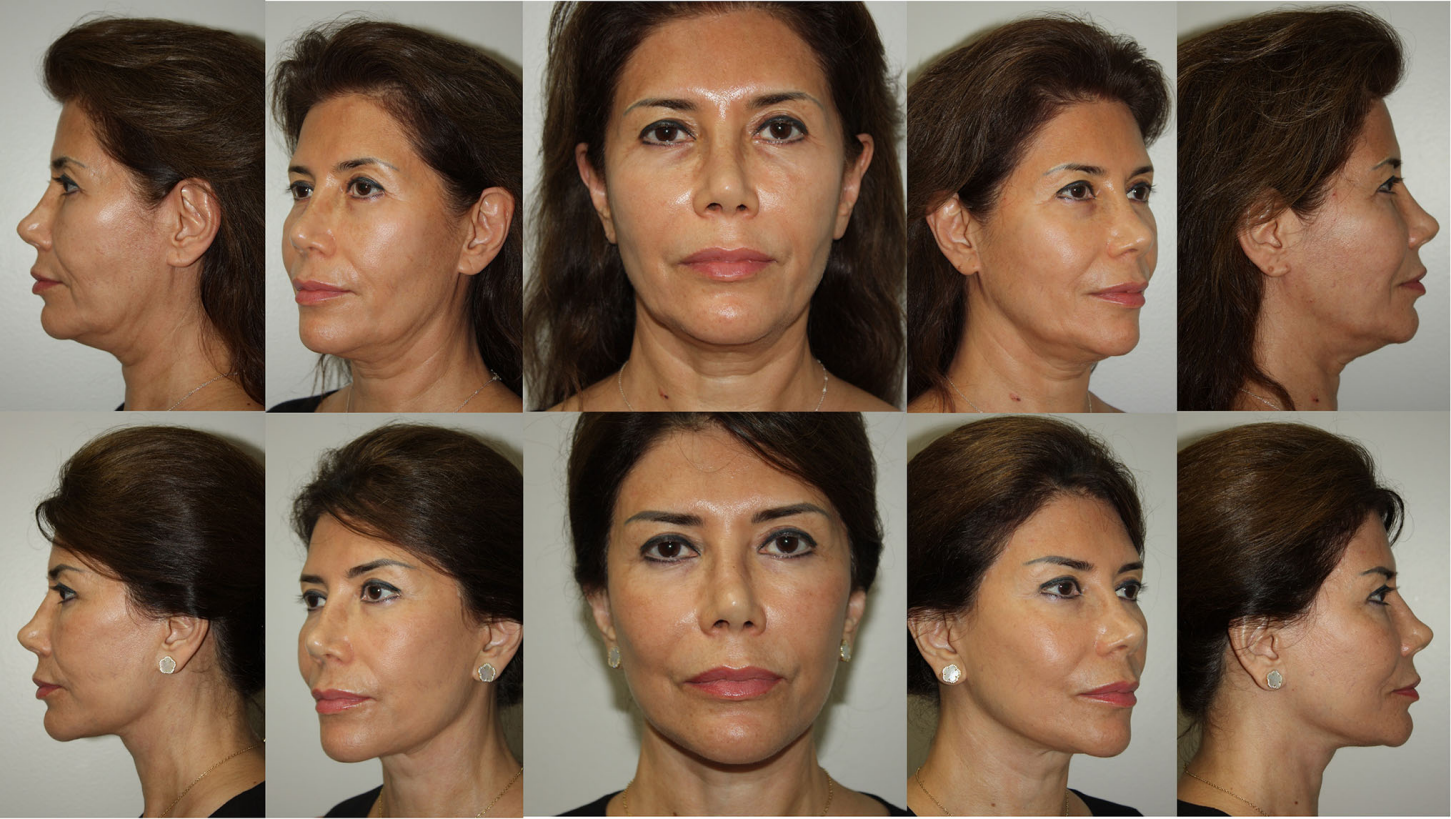
60 year old woman who is 4 years post op from deep plane facelift and endoscopic brow lift only. No other procedures have been performed nor additional volume added

This paper does not address rejuvenation of the neck, as our present aim is to present a safe and reproducible manner of composite cheek elevation. Nevertheless, it should be noted it is a routine part of the procedure to routinely transect the platysma. The authors’ view is that complete transverse transaction of the platysma at the thyroid cartilage level allows for a more robust vertical repositioning of the entire monobloc, the cheek and neck composite tissue complex. This has a salutary effect on cheek rejuvenation, effacement of jowls and the longevity of the results, as exemplified in the representative cases (See Figs. 6, 7, 8, 9).

Our review of 136 (121 females, 15 males) consecutive single surgeon facelift cases, performed over 2.5 years from February 2021 to September 2023, is a testament to the safety of this technique, with no flap necrosis, no infections, one hematoma, and three cases of temporary neuropraxia. The authors believe that controlling postoperative nausea and vomiting (PONV) and postoperative blood pressure contributes greatly to the low incidence of hematoma. We obtain a detailed history of prior anesthesia experience and history of motion sickness, to identify patients that are at high risk for PONV. We coordinate our preoperative protocol with our anesthesia providers, that can include Emend, Scopolamine Patch, Zofran, as well as proper individualized anesthesia management. All patients undergo preoperative medical evaluation, including self-administered blood pressure measurements for 4–5 days preop, that will occasionally uncover occult hypertension. Postoperatively, the patient’s blood pressure is measured every two hours for the first 24 h, and every 4 h for the next 24 h. Any systolic BP reading higher than 125 mm Hg is treated with oral Clonidine. Preoperatively, the patients are treated with an oral medication cocktail consisting of Tylenol 1 gram, Celebrex 400 mg, and Gabapentin 100-300 mg. Every patient gets a custom postoperative pain management regimen. Additionally, we incorporate frequent early patient visits to manage any early healing issues. The detailed step-by step method of dissection and fixation that we describe has allowed us to attain meaningful long-lasting results with low complication rates. See Figs. 6, 7, 8, 9.

## Supplementary Information

Below is the link to the electronic supplementary material.

Supplementary file1 (MOV 46167 KB)

Supplementary file2 (MOV 45101 KB)

Supplementary file3 (MOV 64730 KB)

Supplementary file4 (MOV 11098 KB)

Supplementary file5 (MOV 69305 KB)

Supplementary file6 (MOV 68039 KB)

Supplementary file7 (MOV 60612 KB)
